# Time‐course expression QTL‐atlas of the global transcriptional response of wheat to *Fusarium graminearum*

**DOI:** 10.1111/pbi.12729

**Published:** 2017-04-21

**Authors:** Mina Samad‐Zamini, Wolfgang Schweiger, Thomas Nussbaumer, Klaus F.X. Mayer, Hermann Buerstmayr

**Affiliations:** ^1^ Institute for Biotechnology in Plant Production (IFA‐Tulln) BOKU ‐ University of Natural Resources and Life Sciences Tulln Austria; ^2^ Plant Genome and Systems Biology Helmholtz Zentrum München Neuherberg Germany; ^3^ Present address: BIOMIN Research Center Tulln 3430 Austria; ^4^ Present address: Division of Computational System Biology Department of Microbiology and Ecosystem Science University of Vienna Vienna 1090 Austria

**Keywords:** eQTL, wheat, *Fusarium graminearum*, *Fhb1*, *Qfhs.ifa‐5A*, genetical genomics

## Abstract

Fusarium head blight is a devastating disease of small grain cereals such as bread wheat (*Triticum aestivum*). The pathogen switches from a biotrophic to a nectrotrophic lifestyle in course of disease development forcing its host to adapt its defence strategies. Using a genetical genomics approach, we illustrate genome‐wide reconfigurations of genetic control over transcript abundances between two decisive time points after inoculation with the causative pathogen *Fusarium graminearum*. Whole transcriptome measurements have been recorded for 163 lines of a wheat doubled haploid population segregating for several resistance genes yielding 15 552 at 30 h and 15 888 eQTL at 50 h after inoculation. The genetic map saturated with transcript abundance‐derived markers identified of a novel QTL on chromosome 6A, besides the previously reported QTL *Fhb1* and *Qfhs.ifa‐5A*. We find a highly different distribution of eQTL between time points with about 40% of eQTL being unique for the respective assessed time points. But also for more than 20% of genes governed by eQTL at either time point, genetic control changes in time. These changes are reflected in the dynamic compositions of three major regulatory hotspots on chromosomes 2B, 4A and 5A. In particular, control of defence‐related biological mechanisms concentrated in the hotspot at 4A shift to hotspot 2B as the disease progresses. Hotspots do not colocalize with phenotypic QTL, and within their intervals no higher than expected number of eQTL was detected. Thus, resistance conferred by either QTL is mediated by few or single genes.

## Introduction

Analogous to phenotypic quantitative trait loci (QTL) variation in transcript levels can be mapped to loci governing the expression of underlying genes. Originally termed ‘genetical genomics’ (Jansen and Nap, 2001) expression QTL (eQTL) approaches combined with transcriptome‐wide mRNA abundance measurements allow to generate a detailed picture of changes in the regulatory landscape based on genotypic differences in structured and natural populations. In strongly investigated biological systems such as human (Brown *et al*., 2013; Dixon *et al*., 2007) or *Arabidopsis thaliana* (Lowry *et al*., 2013), full genome sequences and haplotypes for large panels of individuals with reduced linkage disequilibrium can be fully exploited to yield not only genetic loci but causative polymorphisms for mapped eQTL. However, studies in large and complex crop plants such as hexaploid wheat (*Triticum aestivum*) still lack behind, as many of the required genomic resources are only being implemented (Jordan *et al*., 2015; The International Wheat Genome Sequencing Consortium, 2014). Nonetheless, relevant insights on the genetics of gene expression regulation have been acquired from less resolved experiments (Breitling *et al*., 2008; Brem *et al*., 2002; Schadt *et al*., 2005; Yvert *et al*., 2003). The feasibility of eQTL studies in crops to detect causative variants was demonstrated by Druka *et al*. (2008), who successfully correlated the barley (*Hordeum vulgare*) *Rpg1* R‐gene locus to a large effect eQTL corresponding to the cloned stem rust resistance gene *Rpg1* in a segregating biparental population. Similar studies to identify candidate genes or master regulators in crop plants were conducted in barley (Chen *et al*., 2010; Moscou *et al*., 2011), *Brassica rapa* (Hammond *et al*., 2011), maize (*Zea mays*, Shi *et al*., 2007) and rice (*Oryza sativa*, Wang *et al*., 2014). These findings have been expanded by moving from candidate genes to systems biology to describe the biology underlying transcriptional hotspots – loci that govern the expression of hundreds of genes – by illustrating the concerted action and functional similarities of groups of co‐expressed genes therein (Keurentjes *et al*., 2007; Munkvold *et al*., 2009; Wang *et al*., 2014).

The response of wheat to the prevalent pathogen *Fusarium graminearum,* causing Fusarium head blight (FHB), has been extensively investigated on the phenotypic level yielding dozens of QTL in diverse germplasm (Buerstmayr *et al*., 2009). The spectrum of resistant responses is mainly categorized in resistance against initial penetration of the pathogen (type I) and resistance against spreading of the disease (type II) (Schroeder and Christensen, 1963). The complex nature of the underlying resistance mechanisms has been investigated in transcriptomic studies in near‐isogenic material (Hofstad *et al*., 2016; Nussbaumer *et al*., 2015) but not on the population level: After infection *F. graminearum* commences with a biotrophic life style but switches to necrotrophy after roughly 48 h. This switch is set off or followed by the production of high amounts of the trichothecene toxin deoxynivalenol (DON) that elicits oxidative stress and shuts down ribosomal peptidyltransferase activity (Pestka, 2010). These dynamic changes are countered by massive reprogramming of the host transcriptional response (Nussbaumer *et al*., 2015). The outcome of these interactions is much determined by the plants correct response at the right time to counteract the multifaceted effects of DON (Audenaert *et al*., 2014).

Mapping eQTL in a population segregating for FHB resistance seems well suited for observing the transcriptional dynamics underlying these interactions. Here, we captured transcriptome profiles at two time points after inoculation with *F. graminearum* from the CM‐82036 × Remus doubled haploid (DH) population that segregates for two prominent resistance QTL *Fhb1* and *Qfhs.ifa‐5A* (Buerstmayr *et al*., 2002, 2003). Both time points after inoculation depict snapshots before and after the host reaction to DON in near‐isogenic lines derived from the same parental genotypes (Kugler *et al*., 2013). We have explored the population‐wide transcriptomic data to (i) saturate the genetic map with transcript‐derived markers (TDM) yielding a yet undescribed resistance QTL in this population after revisiting previously generated phenotypic data. We (ii) describe eQTL and candidate genes mapping to the intervals of all three QTL and (iii) used a systems biology approach to analyse eQTL accumulated in hotspots at either time point to describe the reconfiguration of the transcriptional landscape as the disease progresses.

## Results

### Improved map resolution yields a novel FHB resistance QTL

Global expression profiles at 30 and 50 h after inoculation (hai) were captured from *F. graminearu*m‐inoculated wheat heads of 163 individuals of the Remus × CM‐82036 DH population including the parents using a custom 8 × 60 k Agilent microarray (Santa Clara, CA). A total of 1500 TDM exhibiting distinct biallelic expression patterns between the parental lines were used in combination with existing SSR and AFLP genotypes (Buerstmayr *et al*., 2002) to generate an improved genetic map of 33 linkage groups including 183 TDMs and 247 other nonredundant marker scores (Table S1). We employed phenotypic data previously published by our group for type II resistance (Buerstmayr *et al*., 2002) and field resistance, assessing primarily type I and to a lesser extent type II resistance by spray inoculation (Buerstmayr *et al*., 2003), as well as resistance again DON (Lemmens *et al*., 2005) and combined these data with new phenotypes for type II resistance obtained in the greenhouse in course of this study for QTL mapping (Table S2). Significant thresholds for logarithm of odds values (LOD) were obtained using simple interval mapping (SIM) and the Haley–Knot regression method with 1 cM intervals (*P*‐value ≤ 0.01, 1000 permutations).

**Figure 1:**
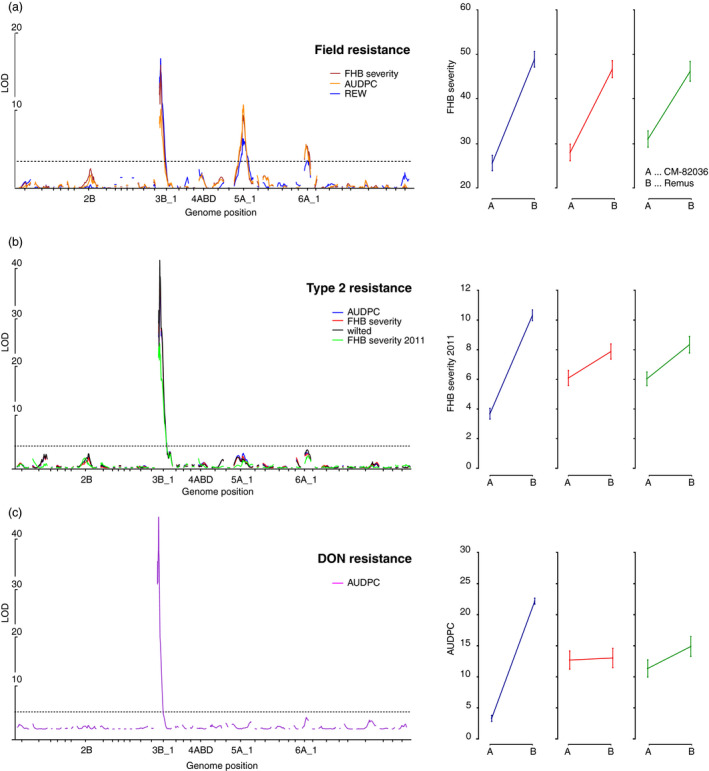
Interval analysis and effect plots of QTL for (a) field resistance, (b) type II resistance and (c) resistance against DON. AUDPC refers to the area under the disease pressure curve, REW to the relative ear weight and FHB severity to the percentage of diseased spikelets. The phenotypes are described in more detail in Table S2.

Using the new saturated map, we detected a novel field resistance QTL on chromosome 6A, placed in a 1.8 cM interval between markers *S1824_1_6A_1* and *A_99_P490642* and confirmed the previously reported strong contributions to type II resistance and DON resistance from *Fhb1* and to field resistance by *Qfhs.ifa‐5A* (Figure 1). The resistant allele for the 6A QTL originated from the *Fhb1* and *Qfhs.ifa‐5A* donor CM‐82036. Similar to *Qfhs.ifa‐5A,* the 6A QTL does not contribute to resistance against DON.

### Establishing the eQTL‐atlas of the wheat response to FHB

15 552 (30 hai) and 15 888 eQTL (50 hai) were generated from normalized expression data of 163 DH lines (SIM, Haley–Knot regression, 1 cM walking steps). Significance thresholds were determined by selecting the 95th percentile from the distribution of the maximum LOD scores (*P*‐value < 0.01, 1000 permutations). The majority of eQTL in each experiment had LOD values between 3 and 6 (48% at 30 hai and 56% at 50 hai) or higher than 10 (31% at 30 hai and 27% at 50 hai, Figure S1a). 40% of the significant eQTL at 30 hai and 32% of eQTL at 50 hai explained 20% or more of the target genes expression variation (Figure S1b). Roughly 82% of all recorded genes are under the control of a single eQTL. About 15% were affected by two eQTL and only a few hundred genes were governed by three eQTL or more (Table S3). To identify segregating expression of genes with changed expression pattern between 30 hai and 50 hai – indicating a changed response to the pathogen in time – we generated eQTL from the differences of normalized, log_2_‐transformed signal intensities recorded at either time point (ΔeQTL = 50–30 hai) yielding 1880 ΔeQTL governing the expression of 2036 genes. Additionally, 370 and 301 differentially expressed genes (DEG) between mock and *F. graminearum*‐inoculated parental lines Remus and CM‐82036 were detected at 30 hai and 4735 and 4272 DEG 50 hai respectively, which reflects the magnitude of recorded expression changes in the ΔeQTL analysis (Table S4).

Most eQTL are not physically located proximal (cis) to the position of the genes under their control. Such trans‐acting eQTL map distal to target genes at the positions of regulatory elements that influence gene expression variation. To establish a robust LOD threshold to differentiate cis from trans‐eQTL, we estimated the genetic positions of 7662 genes corresponding to eQTL at either time point by transposing our map data to the consensus map of the wheat reference cultivar Chinese Spring used the wheat genome zipper (Mayer *et al*., 2011; Figure S2a). Genes mapping within 15 cM of the corresponding eQTL were declared cis‐eQTL. The distributions of recorded cis‐LOD (median 16.48, 30 hai; 13.72, 50 hai) and trans‐LOD (5.3 and 5.1) were used to estimate an arbitrary LOD threshold of 10, to best separate cis‐ and trans‐eQTL (Figure S2b). Applied onto the entire eQTL, data 68.5% of eQTL were thus considered trans‐eQTL at 30 hai and 73.3% at 50 hai, which is in good agreement with reports from literature (i.e. Swanson‐Wagner *et al*., 2009).

### eQTL colocalizing to FHB resistance QTL

eQTL data can be directly explored for (cis) eQTL mapping into phenotypic QTL intervals. Nineteen and 21 eQTL at 30 hai and 50 hai map within a 3 cM interval including *Fhb1* between markers Gwm389 and Barc133 (Table S4). None of these matched the 28 candidate genes located in the recently sequenced interval of *Fhb1* (Schweiger *et al*., 2016), because many of these comprise variety‐unique genes, are poorly expressed and/or are annotated as low confidence genes. Still, trans‐regulated eQTL may be implicated as downstream targets of the QTL activity. The locus does not accumulate a higher than average number of eQTL (see below). Single trans‐eQTL (Table S4) such as a small molecule UDP‐glycosyltransferase (*A_99_P029249*, GenBank accession: *BG274933*) may yet be contributing to metabolize DON (Poppenberger *et al*., 2003).

One hundred and four (30 hai) and 83 (50 hai) eQTL of which 50 are shared between time points map to the 2 cM interval between TDM *A99_P308566* and *Barc141* describing *Qfhs.ifa‐5A* on linkage group 5A‐1 (Table S4). Type I, penetration resistance against initial infection of the pathogen conferred by *Qfhs.ifa‐5A* requires early activation and may be active at both time points. Of the eQTL present at both time points 18 and 26 genes are higher expressed across the population for the susceptible QTL allele or the resistant allele, respectively (Figure S3) but none were also DEG in the parental lines. Thus, constitutively expressed genes need to be considered as candidates: Of these several genes with higher transcript abundance for the resistant allele contain annotations that could explain the QTL activity, including a receptor‐like protein kinase (*A_99_P352346*, tentative consensus ID: *TC433868*), a previously described lipid transfer protein (*CUST_769_PI425885773*,* CA635994*, Schweiger *et al*., 2013) and a XH/XS domain‐containing protein (*CUST_1123_PI425860077*,* BJ274407*) implicated in RNA‐directed DNA methylation (Ausin *et al*., 2012).

Only four (30 hai) and two eQTL (50 hai) are associated to the location of the field resistance QTL identified on chromosome 6A between markers *S1824_1_6A_1* and *c6A_1.loc9* (Table S4). *A_99_P342731* (*TC382014*) encoding a receptor kinase corresponds to an eQTL present at both time points, which may participate in early pathogen recognition. Two eQTL relate to genes encoding aquaporins that regulate water permeability and re‐establish the disturbed plant cell osmotic balance and nutrient homoeostasis in response to stress (Afzal *et al*., 2016).

### Three major eQTL hotspots orchestrate the segregating defence response

eQTL in both experiments were not equally distributed across the genome but formed clusters at several positions. We estimated significant deviations from the expected number of eQTL per chromosome (total eQTL/cM * chromosome length, *P*‐value ≤ 0.0001, chi‐square test). Standardized residuals (SR = (observed–expected/(expected)^−2^) larger than 2.33 indicated significant higher numbers of eQTL than expected (*P*‐value ≤ 0.001) on seven linkage groups at either time points, of which six were detected in both time points. The linkage group comprising chromosome 2B (SR = 45.31) contained higher numbers only in the 50 hai data set and linkage group 2 of chromosome 5B (SR = 2.88) only in the 30 hai data set.

**Figure 2:**
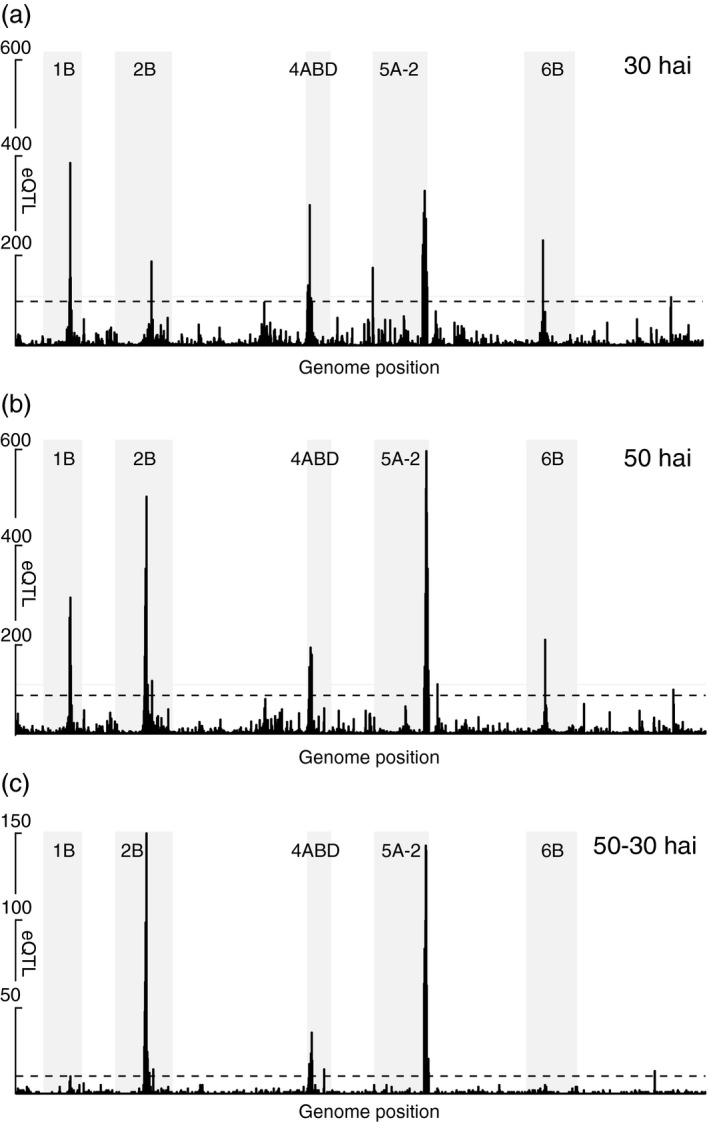
Frequency of eQTL at (a) 30 hai, (b) 50 hai and (c) ΔeQTL sorted along the ordered wheat linkage groups/chromosomes. The green solid horizontal line denotes 100 eQTL for scale, and the red dashed horizontal line indicates the threshold for eQTL hotspots. Shaded areas indicate linkage groups with hotspots.

To determine whether the significantly higher abundance of eQTL originates from regulatory hotspots, the number of eQTL/cM was plotted along the genome (Figure 2) and significance thresholds of 78 (30 hai), 90 (50 hai) and 10 (ΔeQTL) eQTL/cM were established by permutation test from the 99.9th percentile of the sorted, highest recorded eQTL frequencies (1000 permutations). This identified five regions, of which hotspots on chromosome 2B at 50 hai, chromosome 4A and chromosome 5A at both time points show a highly increased ratio of trans‐eQTL (eQTL with LOD < 10) compared to the genome‐wide average.

**Figure 3:**
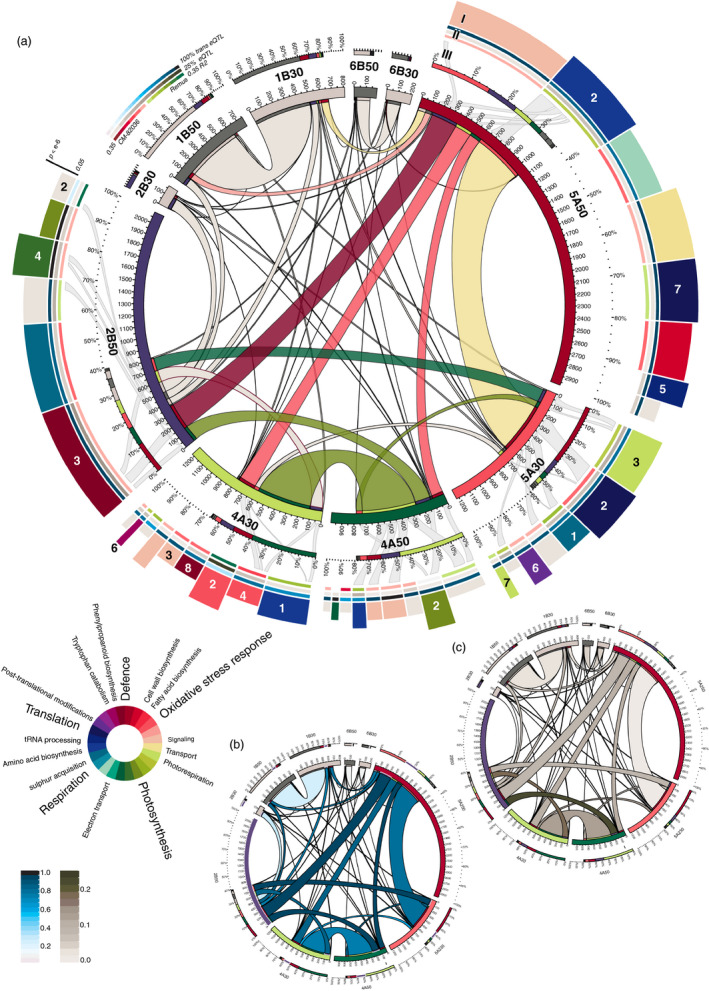
Circular representations of interactions between regulatory hotspots. (a) Segments sizes of the inner ring and ribbon sizes represent the total number of eQTL for each transcriptional hotspots detected at either 30 or 50 hai and shared number of eQTL respectively. Overlaps between these groups of shared eQTL to other groups have not been considered. (I) Sizes of rim segments refer to the number of co‐expressed eQTL in clusters generated for each hotspot. Colours refer to the highest enriched GO terms (circular legend). Clusters numbering corresponds to the original data in Figure S4 and are given only if mentioned in the text. (II) The percentage of trans‐eQTL per cluster, the percentage of ΔeQTL as an indicator of expression difference between time points and the heritability colour‐coded for the allele with relative higher expression across the population are given for each cluster. (III) The localization of ‘shared’ eQTL between hotspots (central ribbons) in distinct clusters is indicated by second‐tier ribbons for selected groups only to reduce complexity. Analogous to (a) but ribbon colours reflect trans‐eQTL ratios (c) and the percentage of eQTL, which are also ΔeQTL for either involved hotspot (c).

Potentially coregulated eQTL in each hotspot were further dissected into groups of co‐expressed genes by hierarchical clustering of the normalized and transformed transcript abundance measurements (Figure S4). Most of these clusters are enriched for eQTL with distinct biological functions based on Gene Ontology (GO) terms (Figure 3a, track I). Yet, these co‐expression clusters do not uniformly show higher expression for a distinct parental allele and allelic effects in each cluster vary largely with a median heritability per hotspot ranging from 0.14 to 0.18 (Figure 3a, track II). To give additional weight to the interpretation of each cluster also trans‐eQTL ratios and ΔeQTL content were established (Figure 3a, track II and Figure 3b and c). All hotspot and cluster‐related data are deposited in Table S5.

### Reconfiguration of regulatory roles during disease development

The hotspot on chromosome 2B gains momentum only at the later time point, where it accumulates more than 2000 eQTL (trans‐eQTL ratio: 0.9) compared to 172 eQTL (trans‐eQTL ratio: 0.5) at 30 hai. While many of the eQTL only come into existence at 50 hai, 200 eQTL on 2B were under control of hotspots on 4A and 5A at the earlier time point (Figure 3a, ribbons). Additionally, 45% of the 1415 genes unique to the 2B hotspot were under the control of non hotspot‐related eQTL at 30 hai spread throughout the chromosomes.

The hotspot is strongly involved in regulating the segregating defence response including the biosynthesis of phenylpropanoids and efflux pumps (cluster 3_2B50, Figure 3a) as well as photosynthesis (4_2B50). Both clusters contain high trans‐eQTL ratios (>0.98) and a higher than average number of eQTL correspond to ΔeQTL in 3_2B50 (14% compared to 8% for the hotspot average), demonstrating an active role in defence response at this time point. Genes in both clusters are highly accessed by other hotspots (Figure 3a, track III).

The hotspot on chromosome 4A controls a higher transcriptional activity at 30 hai (1223 eQTL) compared to 50 hai (991 eQTL). The biological mechanisms controlled at this locus change fundamentally between time points: At 30 hai, clusters are heavily enriched in defence‐related terms, whereas at 50 hai, the highest enriched terms relate to photosynthesis. In contrast to other hotspots, transcript abundances in 4A at 30 hai are strongly affected by the parental allele (Figure 3a, track II and Figure S4). Especially, cluster 3_4A30 (90% trans‐eQTL) has among the strongest allele expression differences and only small variances between the normalized transcript abundances. These tightly controlled genes are higher expressed for the CM‐82036 allele and include transcription factors and kinases relevant for the orchestration of genes in other clusters of 4A encoding for different functions: These are predominantly enriched for fatty acid biosynthesis and oxidative stress reduction terms (4_4A30, biased for CM‐82036 allele), drug efflux (2_4A30, Remus), tryptophan biosynthesis (6_4A30, Remus) and biosynthesis of phenylpropanoids (8_4A30, CM‐82036).

At 50 hai, control of defence‐associated genes has shifted from the hotspot on 4A to 2B. Clear biological implications have only been retrieved for few clusters including 2_4A50 (photosynthesis, no allele preference discernible) of which many genes are coregulated also by the hotspot on 2B.

Clusters in the hotspot on 5A do not seem to be directly involved in defence response but mostly relate to photosynthesis, glycine metabolism and photorespiration (3_5A30), sulphate uptake via cysteine (1_5A30) and translation (2_5A30). Photorespiration is a reoccurring term enriched also in 6_5A30 and 7_5A30), which share an expression bias for the Remus allele. This process does not seem to be retained at 50 hai, as large fractions of the 442 shared eQTL originate from clusters 1_5A30 and 2_5A30.

At 50 hai, almost 3000 regulated genes cover a wide variety of molecular functions. As a common theme, a strong role in translation and protein assembly may be discernible from the cluster enrichments. Genes in cluster 2_5A50 are highly accessed also by other hotspots. It is strongly enriched for amino acid biosynthesis, which also include defence‐related pathways leading to the production of phenylpropanoids. Related functions are enriched in 5_5A50 (tRNA aminoacylation) and 7_5A50 (ribosome biogenesis, tRNA processing).

**Figure 4:**
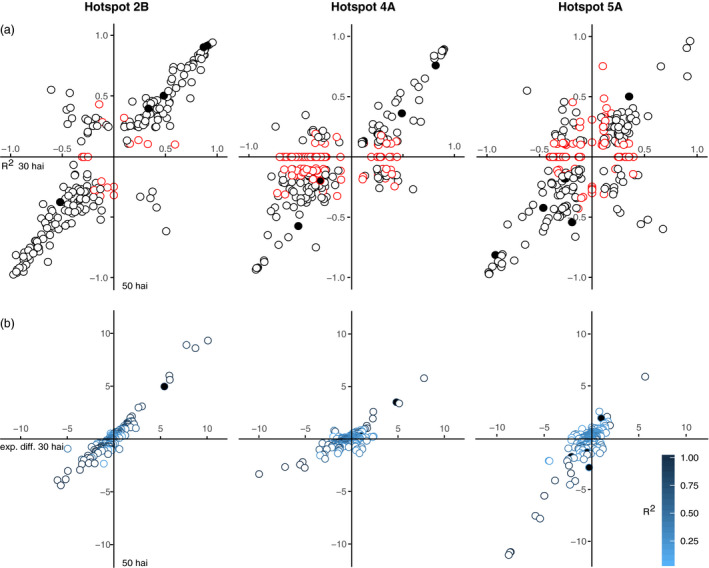
(a) Allelic effect plots for eQTL with LOD > 10 of each hotspot. Red symbols indicate trans‐eQTL mapped to positions other than the respective hotspot at either time point. (b) Expression differences between hotspot eQTL with LOD > 10 grouped by parental allele at 30 hai and 50 hai. Filled symbols refer to eQTL that are also differentially expressed between mock and *F. graminearum* treatment in any or both of the parental lines.

Our findings show that distinct biological mechanisms in single hotspots are higher expressed for different alleles. It is therefore nontrivial to pinpoint strong cis‐eQTL encoded within the hotspot intervals as causal genes based on annotations, allelic effect or LOD values alone. Nevertheless, to limit the range of potential causative genes in each hotspot, we separated eQTL with LOD > 10 by the parental allele providing the higher expression and the heritability observed for this eQTL at either time point (Figure 4a, Table S6) and for expression differences observed for the parents alone (Figure 4b). Cis‐eQTL on hotspot 2B predominantly retain the same allelic effect and heritability between time points (Figure 4a), although the hotspot only gains momentum at 50 hai. Thus these genes are possibly unrelated to the hotspot activity, also none of the eQTL show a striking higher expression difference at the later time point compared to the earlier time point that could be associated with the formation of the hotspot (Figure 4b). For a group of eQTL allele preference changes, predominantly, from the Remus allele to the CM‐82036 allele at the later time point. Also eQTL with large expression differences between parents were only observed with higher transcript abundances for the CM‐82036 allele, which is in line the higher expression for the CM‐82036 allele of genes in most 2B clusters. A gene possibly implicated in the formation of the hotspot is *A_99_P510277* (*R* ^2^ = 0.84 at 50 hai, *TRAES_2B_5E8AC26AF.1*) encoding a protein kinase.

Highly significant eQTL on 4A are fewer in numbers but follow the same trend as observed for 2B. Strikingly, the largest effect eQTL showed reduced expression differences at the later time point, which may directly relate to the dominant defence reaction at 30 hai. Among these, a leucine‐rich repeat protein higher expressed for the Remus allele is prominent candidate (*R* ^2^ = 0.93 at 30 hai, *A_99_P382312*, EST *TA106095_4565*). The strong activity in defence at 30 hai is reflected by a large number of high effect trans‐eQTL at 30 hai. This group is enriched for GO terms relating to oxidative stress reduction and includes P450 monooxygenases and glutathione‐S transferases, efflux pumps and defence‐related transcription factors (Figure 4b). For the majority of these eQTL, the control shifted to the hotspot on 2B at the later time point.

## Discussion

### A map saturated with transcript‐derived markers uncovers a novel field resistance QTL

We first revisited previously published phenotypic data sets for FHB resistance using an updated map saturated with TDM. The increased genetic resolution yielded a novel field resistance QTL positioned on the distal end of linkage group 6A_1. This novel QTL partly explains the higher field resistance of CM‐82036 near‐isogenic lines (NILs) that are lacking both *Fhb1* and *Qfhs.ifa‐5A* in comparison to the susceptible parent Remus (B. Steiner, personal communications). In this region, two other FHB QTL have been previously reported from different donor parents (Anderson *et al*., 2001; Schmolke *et al*., 2005). The QTL on 6A was associated with strong field resistance after spray inoculation, but did not affect resistance to spreading that is assessed by point inoculations. Possibly, the 6A QTL detected by Anderson *et al*. (2001), assessed by counting the number of infected spikelets after point inoculation, relate to the same gene: Their resistance donor parent ND2603 is – just as CM‐82036 – a direct derivative from Sumai‐3, the popular Chinese resistance source.

### *Fhb1* does not elicit a strong transcriptional response

None of the 28 genes encoded in the unique haplotype including *Fhb1* is represented by the microarray, predominantly because these genes are either variety‐unique genes, are poorly expressed and/or annotated as low confidence genes. The recently claimed *Fhb1* gene encoding a ‘pore‐forming toxin’‐like protein (Rawat *et al*., 2016) is no exception as it is only little expressed gene and unique for the *Fhb1* region (Schweiger *et al*., 2016). Rawat *et al*. (2016) suggest that it acts in recognition of fungal carbohydrates and exerts a toxic effect to the fungus. This activity of a sole gene would explain why our study did not detect a larger group of trans‐eQTL mapped to this locus. The proposed *Fhb1* gene however does not explain resistance conferred to DON alone, which most likely is encoded by a second gene in the same region, which includes both traits (Schweiger *et al*., 2016). Again, we detected no direct downstream activity such as the higher expression of genes causing the toxins efficient metabolization such as gluco‐conjugation (Poppenberger *et al*., 2003), and thus also these proposed mechanisms need to be reconsidered.

We propose several candidates that may encode *Qfhs.ifa‐5A*. However, these have to be treated with care: The QTL resides in the pericentromeric region of chromosome 5A, and the chromosomal interval might include many genes. To narrow down candidates for this QTL, other technologies such as deletion mapping of irradiated hybrids (Riera‐Lizarazu *et al*., 2008) might be more suitable than approaches relying on meiotic recombinations. Also *Qfhs.ifa‐5A* does not overlap with loci governing large transcriptional changes. Similar observations have been made before: no regulatory hotspot mapped to the position of the qualitative resistance locus Rpg‐TTKSK on chromosome 5H of barley (Moscou *et al*., 2011) or on one of the several *Puccinia hordei* resistance genes in a different study on barley (Chen *et al*., 2010).

### Highly dynamic eQTL landscape in disease development

The eQTL landscape recorded at both time points is highly different. About 40% of all eQTL at either time point were unique, and more than 2000 of the 9323 eQTL detected at both time points map to different linkage groups. Also transcript abundance levels change in time. These have been mapped by 1800 ΔeQTL of which a majority accumulate to the positions of three large regulatory hotspots. The observed differences between experiments may also be attributed to other factors than genetic control of transcription levels in response to the pathogen: Despite controlled light conditions, an average 4 hour difference between sampling time points will affect the expression levels of genes associated to the circadian clock. Yet, even these do not suffice to explain the massive concerted reconfiguration of genetic control of genes encoding similar functions between hotspots (Figure 3): For instance, the hotspot on 2B governing the expression of more than 2000 genes is only formed at 50 hai. It takes control not only of genes for which no eQTL has been detected at the earlier time point, but also seizes control of about 200 genes regulated by other hotspots at the earlier time point and many more eQTL unrelated to any hotspot. Similar reports exist for barley, where a single large hotspot taking control of eQTL from several loci after inoculation with *Puccinia graminis* has been described (Moscou *et al*., 2011). Large scale reconfigurations following different treatments have also been described in a RIL population of *Brassica rapa* following calcium or magnesium treatment compared to mock (Graham *et al*., 2014).

### Regulatory hotspots

Two hotspots on chromosomes 1B and 6B were excluded from further analysis: both lack the typical high trans‐eQTL content and likely represent recombination‐poor regions: the large putative hotspot on 1B corresponds to the widely used 1BL/1RS wheat/rye translocation (Rabinovich, 1998) present in many CIMMYT‐derived lines such as CM‐82036, which does not recombine with the respective region on 1B of Remus. The remaining three regulatory hotspots govern the expression of thousands of genes. These may have been formed as the consequence of large pleiotropic effects of a single regulatory gene or by separate coupled loci or polymorphisms in shared cis‐regulatory elements of genes controlling different traits (Wagner and Zhang, 2011). Pleiotropy is a common observation in QTL mapping (Yan *et al*., 2011) and pleiotropic effects manifesting as regulatory hotspots at the positions of phenotypic QTL or strong cis‐acting eQTL have been frequently described (Fu *et al*., 2009; Keurentjes *et al*., 2007). Highly pleiotropic genes are under the effect of purifying selection, thus these are less likely to acquire polymorphisms that confer strong resistant phenotypes as these may perturb the delicate balance of the pleiotropic mechanisms. Thus their effect sizes on transcript abundance variations are limited, yet some minor phenotypic effects were recorded at the position of hotspots on chromosomes 2B and 4A (Figure 1) that are both enriched for defence‐related GO terms. Apparently, these are part of a general response mechanism and may be differentially mounted as a consequence of differences in disease progression between resistant and susceptible lines, while contributing little to the overall resistance: Susceptible NILs without *Fhb1* and *Qfhs.ifa‐5A* showed a strong transcriptional response to the pathogen in contrast to their resistant sister lines, which did not require to activate these genes (Schweiger *et al*., 2013).

In our analysis, clustered transcript abundances showed a directional bias towards one parental allele with highly varying effect sizes of the respective alleles on the variation of transcript abundances. Nevertheless, not all clusters in each hotspot followed the same haplotype bias. The extent of allelic dominance is hotspot specific: Potokina *et al*. (2008) found higher expression for one parental allele to range between 56% and 90% in hotspots detected in biparental population of barley. The study of Li *et al*. (2013) showed strong haplotype bias for nine of 10 hotspots with at least 78% of the genes higher expressed for one parent. A more balanced distribution would suggest that the hotspot composition is a result of linked polymorphisms affecting unrelated traits. This issue needs also to be strongly considered when interpreting the biological implications of the observed hotspots.

The results summarized in Figure 3 imply two central mechanisms enriched in the hotspot on 4A: Defence‐related genes at 30 hai with a haplotype bias predominantly for CM‐82036 and secondly genes relating to respiration and amino acid biosynthesis at 30 hai biased for the Remus allele. A third group strongly coregulated by the hotspot at 2B at 50 hai relates to photosynthesis. Within the 2B hotspot, all clusters with significant functional enrichments showed a haplotype bias for the CM‐82036 allele including the respective photosynthesis clusters. These separate mechanisms seem to support the linkage model. Photosynthesis‐related genes might also segregate independently of disease development. Large genotype‐dependent expression differences for photosynthesis‐related genes have also been identified in a recent eQTL study on tomato (Ranjan *et al*., 2016). Nonetheless, the formation of all three mechanisms may be the consequence of concerted action in response to the pathogen: We have previously demonstrated the extensive effects of *F. graminearum* infection on the primary metabolism, leading to elevated respiration rates and increased amino acid biosynthesis in NILs derived from this DH population (Nussbaumer *et al*., 2015). Also photosynthesis is directly negatively affected by pathogen stress (Berger *et al*., 2007). We could confirm this in detail by comparing our findings to the published data from Nussbaumer *et al*. (2015): Wherein photosynthesis‐related genes (GO:0015979) are less expressed in response to the pathogen compared to mock, while this effect was stronger in the susceptible genotypes.

In contrast, clusters of the hotspot on chromosome 5A did not exhibit a trend for general higher expressed for one parental allele. These clusters also show enrichments for more diverse mechanisms than on the other hotspots. eQTL therein at first seem unrelated to defence, because the large section of eQTL retained between time points – representing a ‘core’ functionality – contained only a small number of ΔeQTL compared to retained eQTL in the 4A hotspot. Nonetheless, this hotspot seems to play an essential role in the hosts’ reaction to DON:

*Fusarium graminearum* switches from a biotrophic to a nectrotrophic lifestyle coincides with the production of large amounts of DON (Audenaert *et al*., 2014) about 48 h after inoculation (Pritsch *et al*., 2000). DON acts mainly via the inhibition of protein biosynthesis by blocking the ribosomal peptidyltransferase centre (Pestka, 2010), causing a variety of resistance‐unrelated reactions such as increased ubiquitination to remove unfinished peptide chains (Lucyshyn *et al*., 2008) and increased biosynthesis of amino acids and tRNA ligases possibly to maintain translational activity (Nussbaumer *et al*., 2015; Warth *et al*., 2014). Corresponding GO terms were enriched at 50 hai in clusters of hotspot 5A. DON also elicits the production of reactive oxygen species (ROS, Nobili *et al*., 2014). Segregating reaction to oxidative stress was recorded at 30 hai on hotspot 4A but also on 5A at 30 hai for terms relating to photorespiration, which also acts in the metabolism of ROS (Sørhagen *et al*., 2013).

Taken together, our analysis illustrates massive genome‐wide transcriptional changes of the host to adapt to the pathogen, which itself transforms from a biotrophic to a necrotrophic lifestyle. We highlight three master regulatory hotspots that orchestrate the expression of thousands of eQTL. Their composition is highly dynamic in time and the control of major defence mechanisms and other processes switches between loci as the disease progresses. To further describe these hotspots, future work needs to be addressed by technologies that use a complete reference genome for RNAseq mapping and high density SNP maps combined with at best large unstructured populations providing low enough linkage disequilibrium to limit the list of causative candidate genes and better resolve the underlying biology. Additionally, mutant lines from the proposed cis‐regulatory genes may be directly characterized on the transcriptional and phenotypic level.

## Experimental procedures

### Plant material and greenhouse experiments

Vernalized seed of 190 individuals from the spring wheat CM‐82036 × Remus DH population (Buerstmayr *et al*., 2002) and parental lines were germinated and vernalized for an additional 3 weeks (4 °C, 12 h light/dark regime) before being transferred to the greenhouse. Plants were potted in a mixture of compost, sand chalk and peat.

The experimental design was a completely randomized design. Each line was planted in an individual pot (with two seedlings) and in five replications (pots). Greenhouse temperatures were gradually increased from 15 °C/13 °C during day/night to 20 °C/18 °C and a 16 h/day photoperiod at the time of anthesis.

Five heads per genotype and sampling time point were required for RNA samples, and 10 heads per genotype were phenotyped to validate the strong segregation for *F. graminearum* resistance in the population. To handle more than 3000 individually treated heads, the experiment was split and conducted in two consecutive greenhouse trials in 2010 and 2011. Sixteen common DH lines were used as replicates in both experiments as well as the parental lines. Each experiment comprised an equal number of lines that included either, none or both of the previously reported resistance QTL, *Fhb1* and *Qfhs.ifa‐5A*.

*Fusarium graminearum* macroconidia spores were isolated from mung bean medium, eluted in water and stored at −80 °C. For each day of inoculation, aliquots of spores were thawn and diluted to 50 000 conidia/mL. Spore germination rates were tested prior each experiment. Six central spikelets on each flowering wheat head were inoculated with 10 μL spore suspension for subsequent RNA extraction. Tissue was collected from the inoculated material as described in Schweiger *et al*. (2013) at 2 pm for samples taken 30 hai and at 10 am for samples taken 50 hai, shock‐frozen in liquid nitrogen and stored at −80 °C for further processing. For phenotyping, two central spikelets were point inoculated. Mock inoculations were only used for parental lines. FHB severity was recorded at 10, 14 and 21 days after inoculation.

### RNA extraction, microarray hybridization and data processing

RNA was extracted from 100 mg of ground tissue comprising samples from five pooled heads using the RNeasy plant mini kit (Qiagen, Venlo, The Netherlands) and checked on a Bio‐Rad Experion automated electrophoresis unit (Hercules, CA). Gene expression profiles were captured using a custom‐built 8 × 60 K one‐colour microarray design based on the Agilent microarray platform (design ID 031677, Agilent, Santa Clara, CA). The design includes the company's commercial 4 × 44 k wheat gene expression array and about 1500 probes that have been designed based on ESTs previously reported as responsive to *F. graminearum* (Bernardo *et al*., 2007; Golkari *et al*., 2007, 2009; Han *et al*., 2005; Hill‐Ambroz *et al*., 2006; Jia *et al*., 2009; Kruger *et al*., 2002; Li and Yen, 2008; Schweiger *et al*., 2013; Walter *et al*., 2008). Sample preparation, hybridization to microarrays and scanning were performed at the facilities of the Austrian Institute of Technology (AIT, Vienna, Austria). Genotypes of which one or both arrays at either time point showed poor properties in the Agilent quality report were excluded from subsequent analysis resulting in 163 samples including the parents for the eQTL analysis and additional three arrays for each parent treated either with mock or *F. graminearum*. Raw data were corrected for background noise and normalized using the R bioconductor/limma package (R Development Core Team, 2008; Ritchie *et al*., 2015).

Alignment software BLAT (Kent, 2002) was used to align ESTs corresponding to microarray probes to the IWGSC wheat genes (version 2.3) by considering the best scoring matches only. GO terms from these data were used for enrichment analysis using R bioconductor package GOstats (Falcon and Gentleman, 2007). To obtain functional descriptions for EST sequences, we first determined putative open reading frames (ORFs) using the TRANSDECODER software version 2.0.1 (https://transdecoder.github.io/) with a minimum ORF length of 240 bp. A total of 74,918 putative ORFs were identified and used as input for the AHRD tool release version 2.0 (https://github.com/groupschoof/AHRD).

### Linkage mapping

A genetic map using 161 DH lines was generated with CarthaGène (de Givry *et al*., 2005) using an existing set of 247 AFLP and SSR marker scores and 183 TDM relating to biallelic expression patterns of single genes across the population. To generate TDMs, the normalized expression values for across all genotypes and parents were clustered by *k*‐means clustering using the R package fpc and validated the coherence of these clusters by *Z*‐test (alpha = 0.001). Genotypes that did not match this criterion were treated as missing data. A maximum distance of 30 cM and a minimum LOD threshold of 3 were used to partition markers into linkage groups. The most likely positions of the markers along the linkage groups were determined using the commands nicemapl, mfmapl, flips, build and annealing in CarthaGène. Genetic distances in cM were generated with the Kosambi function. Markers with less than 0.6 cM distance were merged and the resulting linkage groups compared to consensus maps deposited at Graingenes (http://wheat.pw.usda.gov/GG2/).

### Phenotypic QTL mapping

Published phenotypic data were analysed for QTL using the functions calc.genoprob and sim.geno from the R/qtl package (Broman *et al*., 2003) with 1 cM stepping distance and 16 simulated replicates. Interval mapping was performed using the Haley–Knott regression model (Haley and Knott, 1992). The scanone function was used to determine LOD significance thresholds for type I error (*P*‐value ≤ 0.01, 1000 permutations).

### Differential gene expression analysis

Differentially expressed genes (DEG) for parental lines CM‐82036 and Remus were generated using the eBayes function of the R bioconductor package limma from three replicates of each line and treatment (mock and *F. graminearum*) sampled during the 2012 trial. The false discovery rate was controlled at an adjusted *P*‐value of 0.05 (Benjamini and Hochberg, 1995) and an arbitrary fold‐change cut‐off of 2 was chosen to select more strongly changed DEG.

### Expression QTL mapping

Simple interval mapping (SIM) was performed using R/qtl, and the same parameters for imputing marker data and regression analysis have been employed as described in the phenotypic QTL mapping section. All 163 lines from both trials in 2010 and 2011 were combined in one analysis. A year‐effect was considered by adding a cofactor for year in the regression model (`addcovariate' in the scanone function of R/QTL). A genome‐wide significance threshold for eQTL was determined by selecting the average 95th percentile generated from the representative null distribution of the maximum LOD scores (*P*‐value ≤ 0.01, 1000 permutations). Three individual eQTL mappings were carried out for the data sampled at 30 and 50 hai as well as for a data set generated by subtracting the 30 hai probe signal intensities from the 50 hai probe signal intensities to identify genes showing expression differences between the two time points after *F. graminearum* inoculation across the population (ΔeQTL = 50–30 hai).

Expression profiles of genes under control of the same regulatory hotspot have been clustered using the ‘dist’ function in R for hierarchical clustering including parameter methods ‘Euclidian’ and ‘Ward's’ to generate the distance matrix. The scaled expression values (mean=0; standard deviation=1) of each eQTL were used as independent variables. An additional factor was included to correct for a ‘year’ effect.

## Author contributions

Microarray design, data analysis and interpretation, manuscript writing: WS. Greenhouse inoculations, phenotyping, microarray analysis, phenotypic and expression QTL: MS‐Z. Mapping to IWGSC reference and annotations: TN. Funding and project design: KFXM, HB.

## Supporting information


**Figure S1** Distribution of LOD scores and heritabilities for recorded eQTL at 30 and 50 hai.

**Figure S2** Plotted genetic positions of eQTL and target genes.

**Figure S3** Hierarchical clustering of eQTL mapped to the *Qfhs.ifa‐5A* region.

**Figure S4** Hierarchical clustering of eQTL expression profiles for hotspots on 2B, 4A and 5A.

**Table S1** Genotypes and marker distances for the Remus × CM‐82036 DH population.

**Table S2** Significant LOD for FHB field resistance, type II resistance and DON resistance QTL.

**Table S3** Distribution of eQTL per target gene.

**Table S4** eQTL colocalizing with phenotypic QTL.

**Table S5** Hotspot‐related eQTL, cluster and GO data.

**Table S6** Heritabilities and expression differences for hotspot cis‐eQTL.
