# Supplementary material for: Time‐course expression QTL‐atlas of the global transcriptional response of wheat to Fusarium graminearum
Source: Plant Biotechnol J. 2017 Apr 21;15(11):1453–64. doi: 10.1111/pbi.12729 (PMC5633761; doi:10.1111/pbi.12729)
Supplement: Supplementary file 1 — Figure S1 Distribution of LOD scores and heritabilities for recorded eQTL at 30 and 50 hai. [file PBI-15-1453-s010.pdf]

A

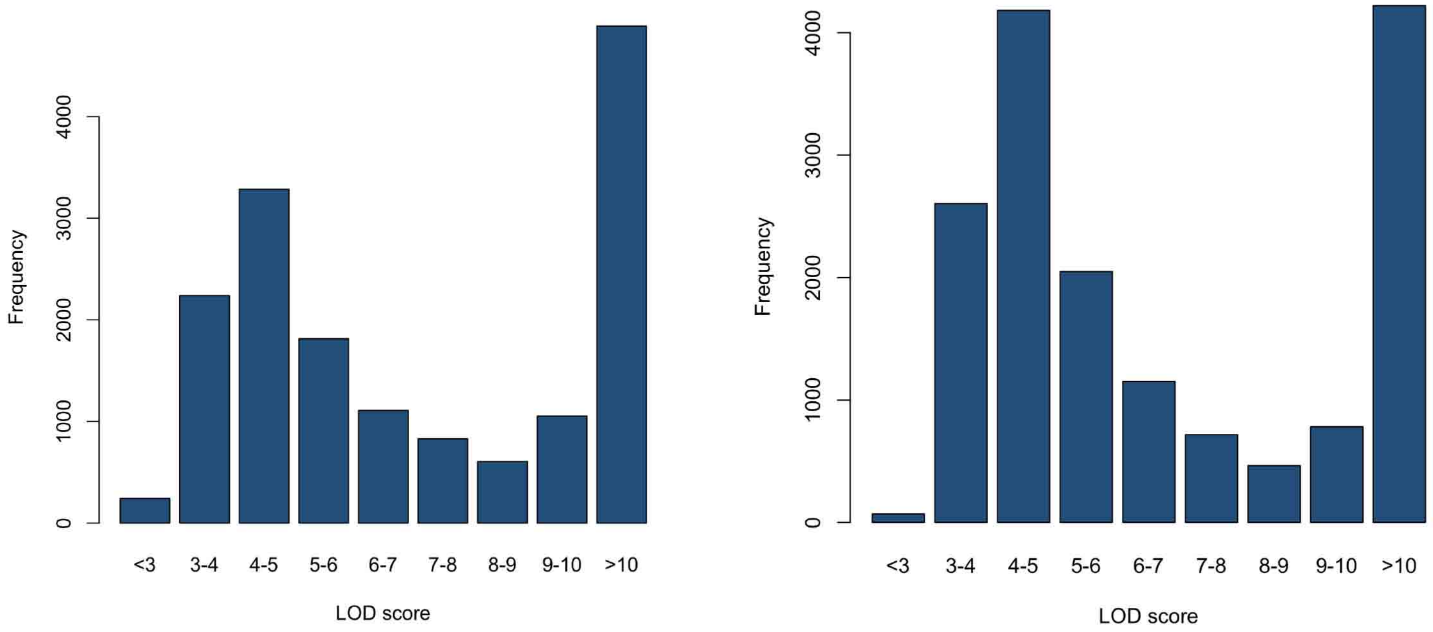

B

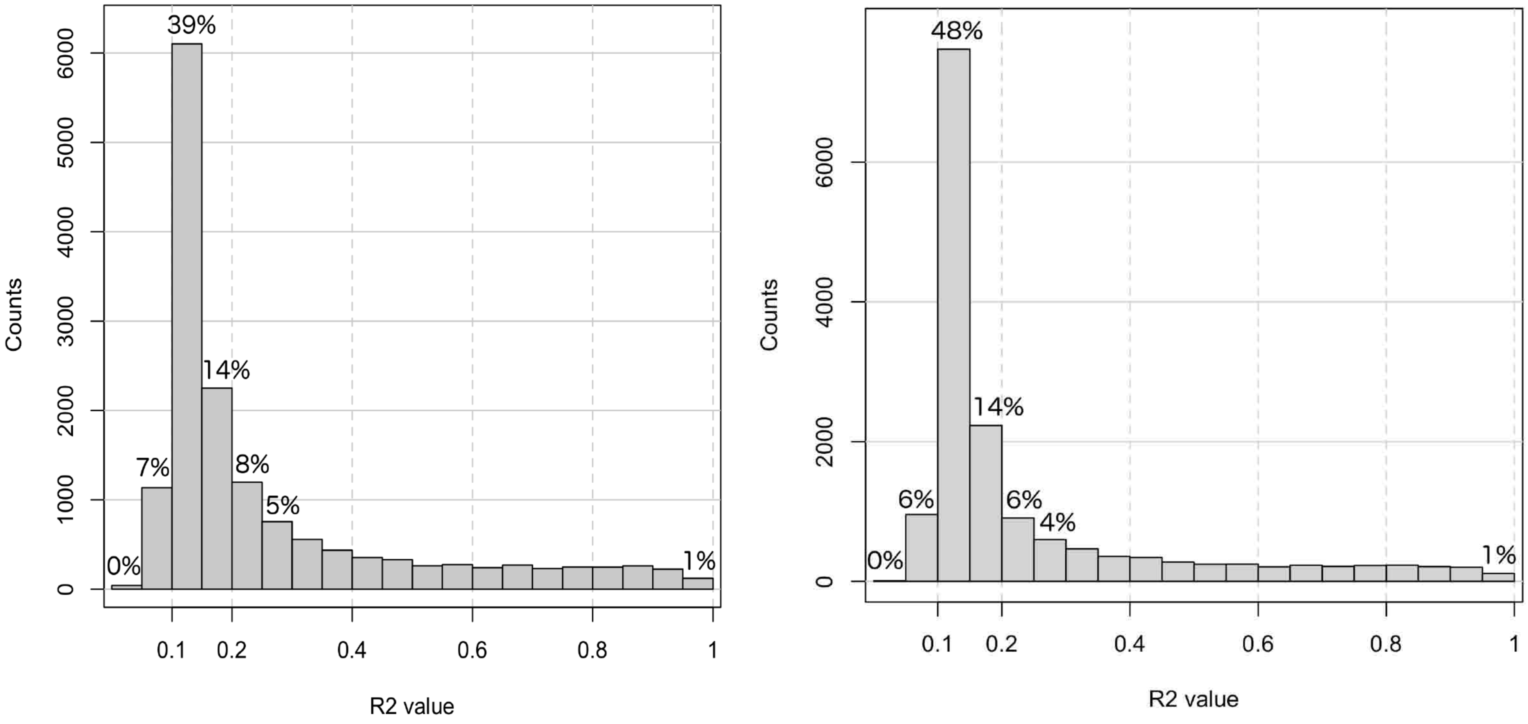

Figure S1. (A) LOD scores for recorded eQTL at 30 hai (left) and 50 hai (right).
(B) Heritabilities (R2) of eQTL at 30 hai (left) and 50 hai (right). Percentages indicate the relative number of eQTL. F.i. 14% of the eQTL at 30 hai explain between 15% and 20% of the expression variation observed for the genes under their control.
