# Supplementary material for: Time‐course expression QTL‐atlas of the global transcriptional response of wheat to Fusarium graminearum
Source: Plant Biotechnol J. 2017 Apr 21;15(11):1453–64. doi: 10.1111/pbi.12729 (PMC5633761; doi:10.1111/pbi.12729)
Supplement: Supplementary file 2 — Figure S2 Plotted genetic positions of eQTL and target genes. [file PBI-15-1453-s009.pdf]

(a)

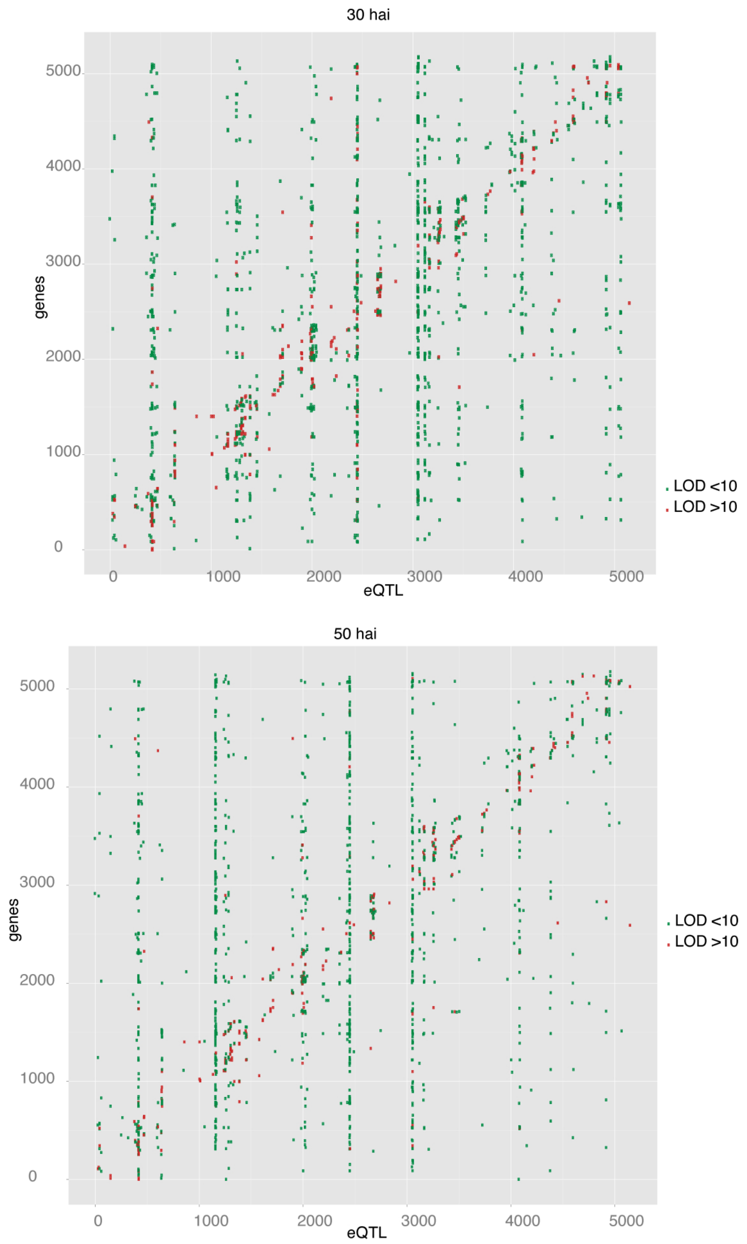

(b)

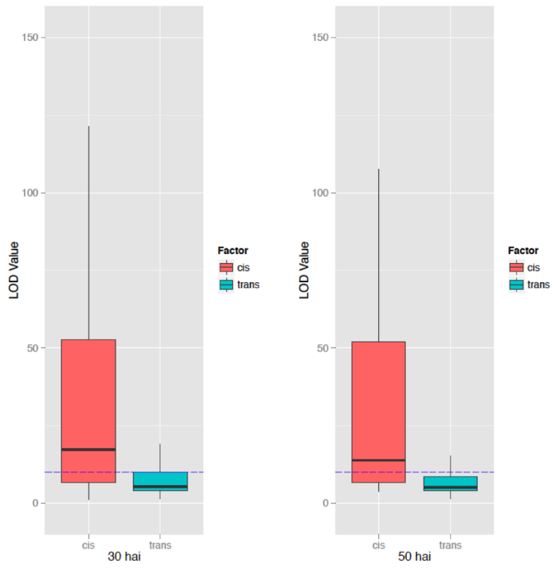

Figure S2 (a) Genome Zipper mapped eQTL position at 30 hai and 50 hai.
x-axis indicates genetic positions of eQTL sorted along the transformed marker intervals from the transposed CM-82036 x Remus map to the Chinese Spring consensus map. Chinese Spring mapped genes are plotted
Green and red dots represent eQTL with LOD score under or above 10, respectively.
(b) Boxplot with LOD distributions of cis and trans mapped eQTL. The dashed indicates a LOD of 10, which was chosen as a cut-off to distinguish cis and trans eQTL in the complete eQTL dataset.
