# Supplementary material for: Time‐course expression QTL‐atlas of the global transcriptional response of wheat to Fusarium graminearum
Source: Plant Biotechnol J. 2017 Apr 21;15(11):1453–64. doi: 10.1111/pbi.12729 (PMC5633761; doi:10.1111/pbi.12729)

Supplementary Figure S3. Hiearchical clustering of eQTL mapped to the *Qfhs.ifa-5A* region. Each Boxplot comprises expression values of grouped eQTL. Boxplots are color coded for presence or absence of the QTL in the respective line. (A) represents eQTL recorded at 30 hai and (B) for 50 hai.

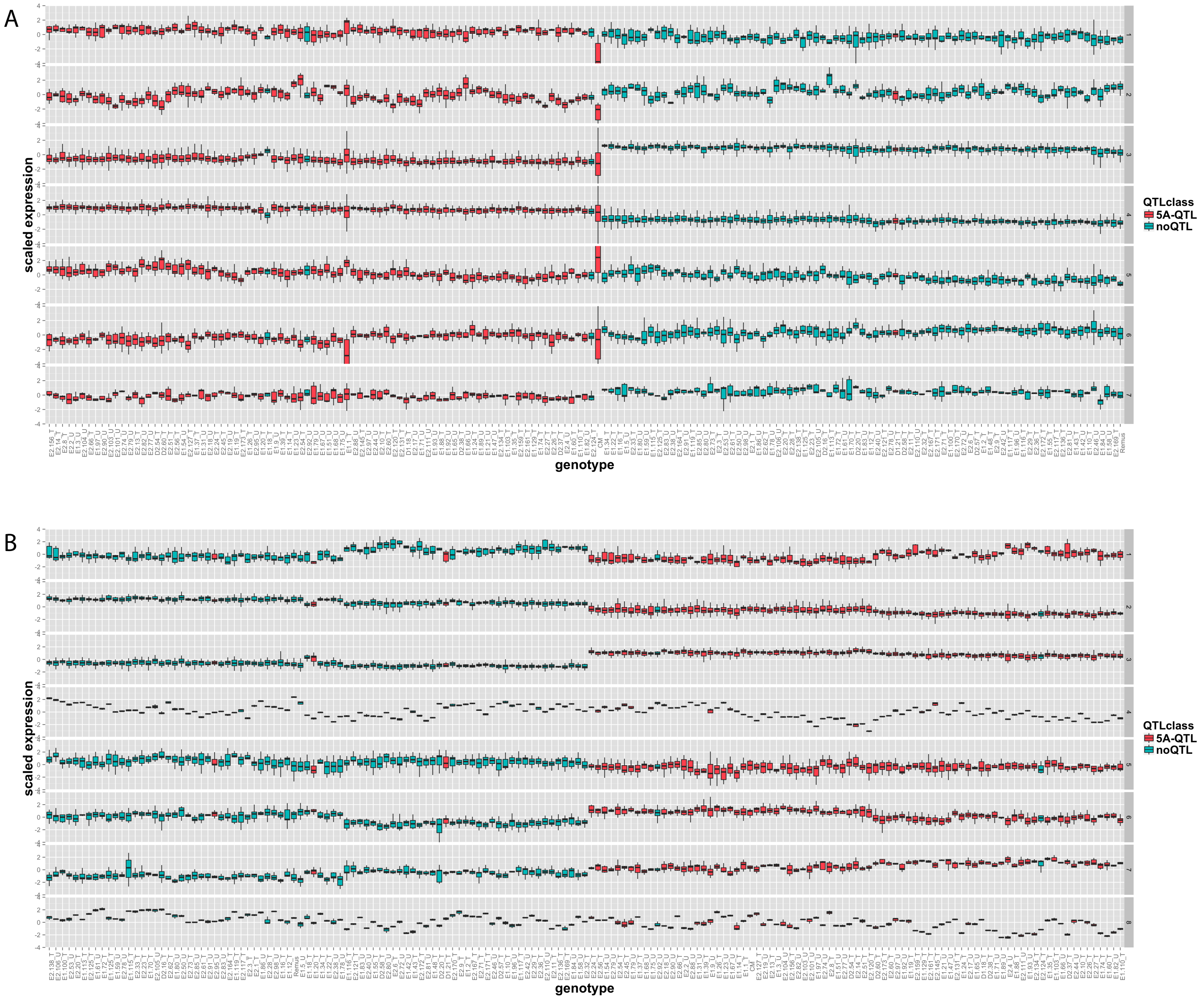

Supplement: Supplementary file 3 — Figure S3 Hierarchical clustering of eQTL mapped to the Qfhs.ifa‐5A region. [file PBI-15-1453-s008.pdf]
