# Supplementary material for: Time‐course expression QTL‐atlas of the global transcriptional response of wheat to Fusarium graminearum
Source: Plant Biotechnol J. 2017 Apr 21;15(11):1453–64. doi: 10.1111/pbi.12729 (PMC5633761; doi:10.1111/pbi.12729)
Supplement: Supplementary file 4 — Figure S4 Hierarchical clustering of eQTL expression profiles for hotspots on 2B, 4A and 5A. [file PBI-15-1453-s007.pdf]

Figure S4 Hierarchical clustering of eQTL expression profiles for hotspots 2B at 30 hai (page 2), 2B at 50 hai (page 3), 4A at 30 hai and 50 hai (pages 4 and 5) and 5A at 30 hai and 50 hai (pages 6 and 7). Parental genotypes (A = CM-82036, B = Remus) at the locus are represented in color for each line. The plotted signal intensities have been normalized and transformed prior clustering.

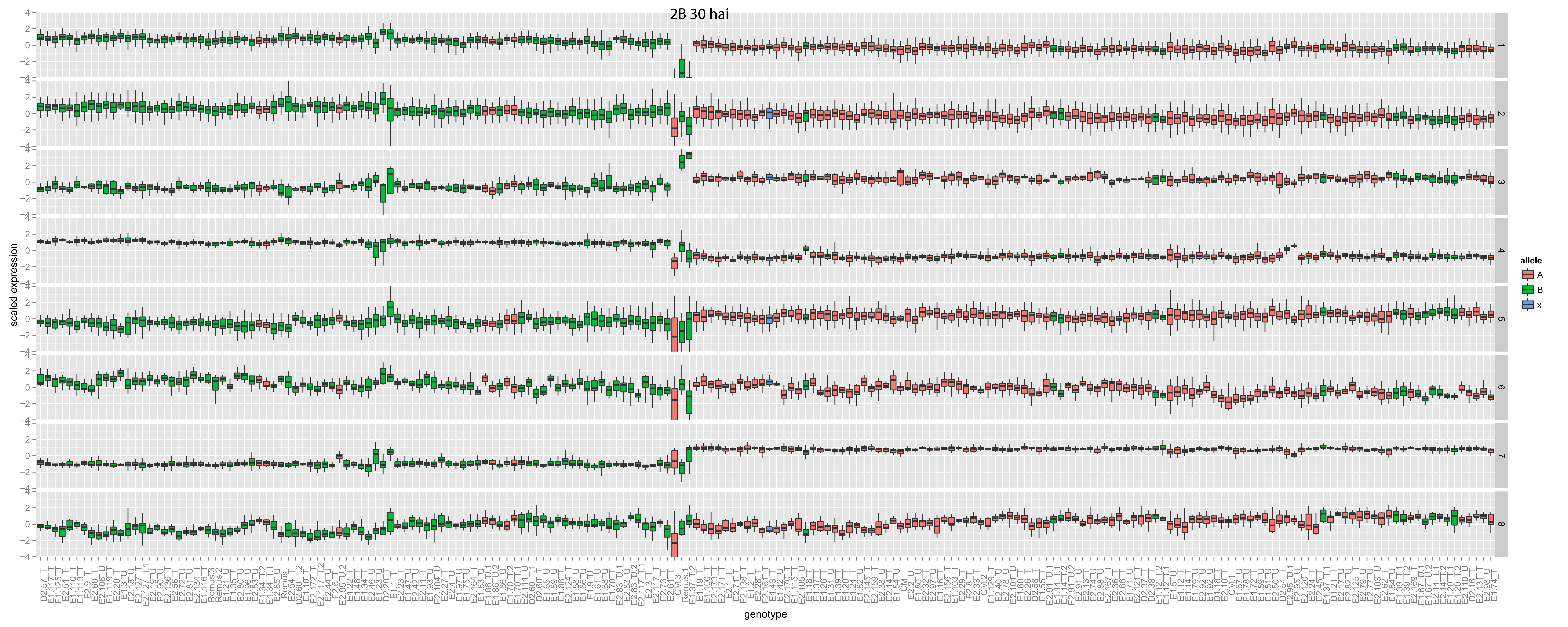

2B 30 hai
scaled expression
genotype
allele
A
B
x

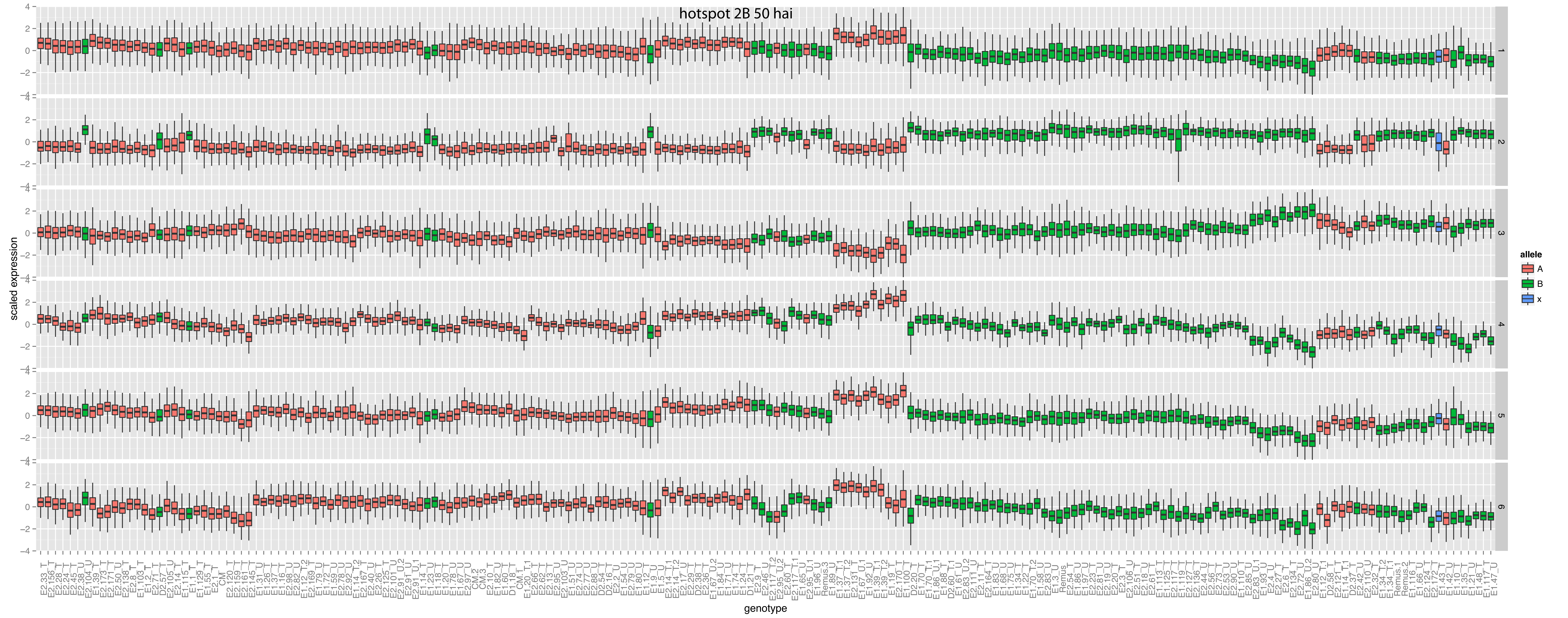
hotspot 2B 50 hai
scaled expression
genotype
allele
A
B
x
1
2
3
4
5
6

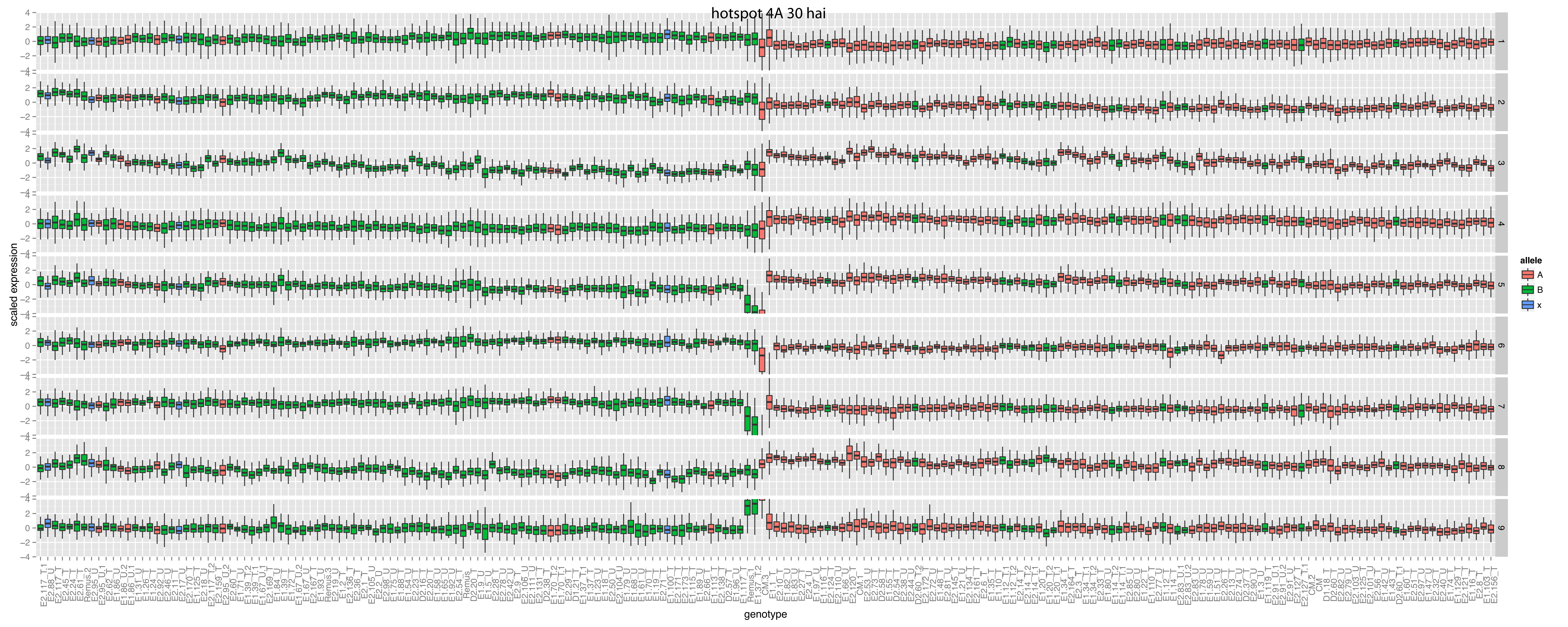
hotspot 4A 30 hai
scaled expression
genotype
allele
A
B
x

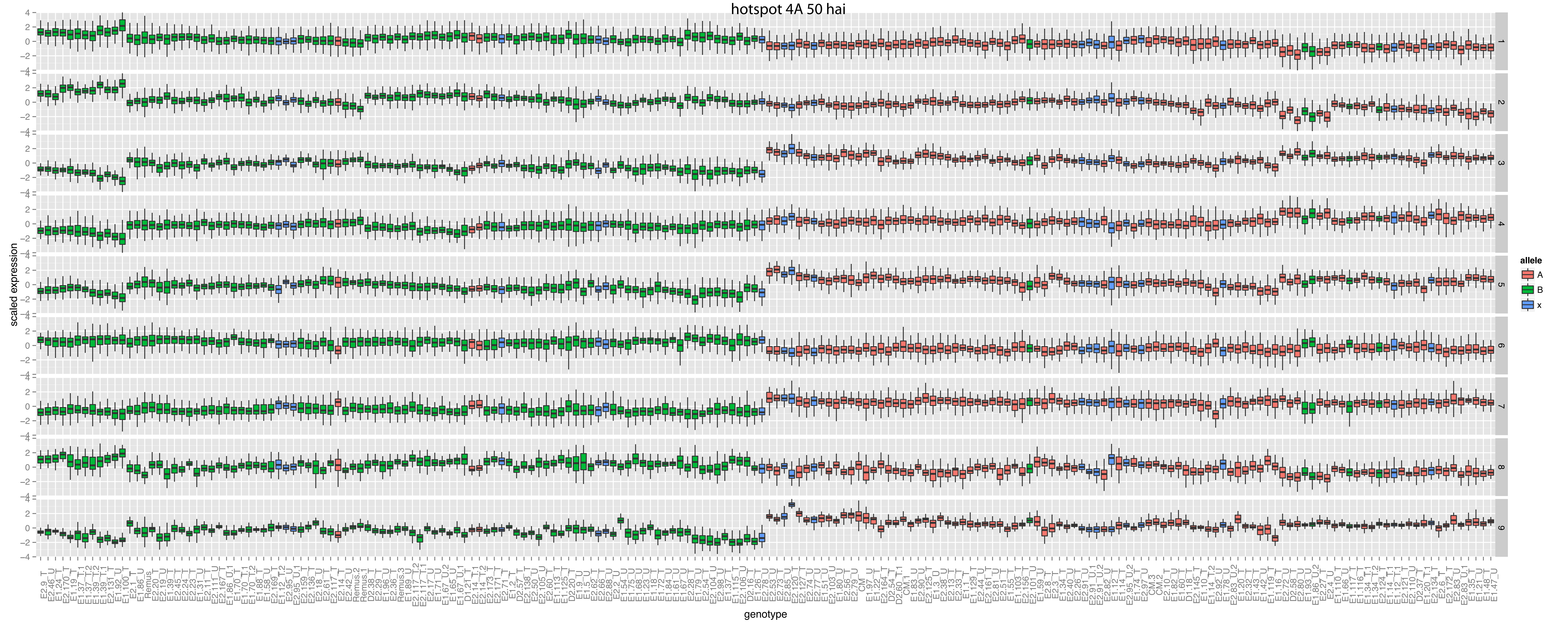
hotspot 4A 50 hai
scaled expression
genotype
allele
A
B
x
1
2
3
4
5
6
7
8
9

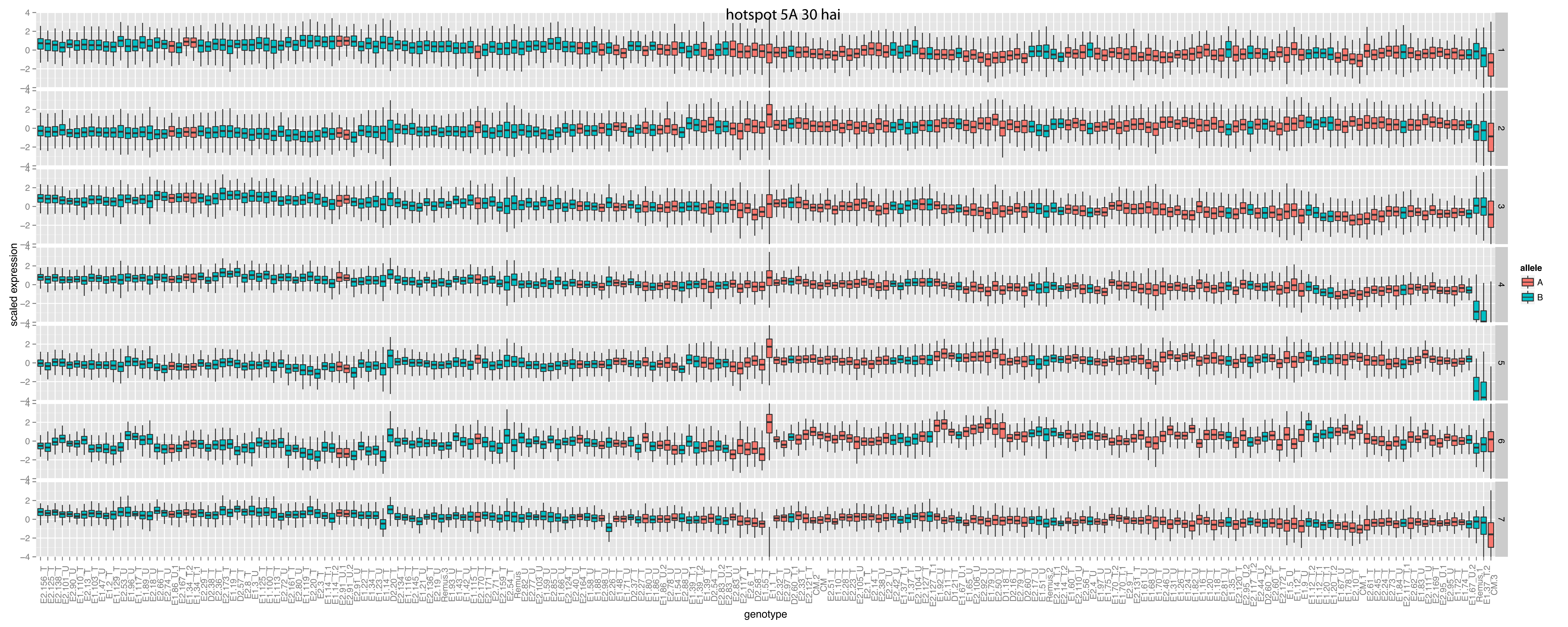
hotspot 5A 30 hai
scaled expression
genotype
allele
A
B
1
2
3
4
5
6
7

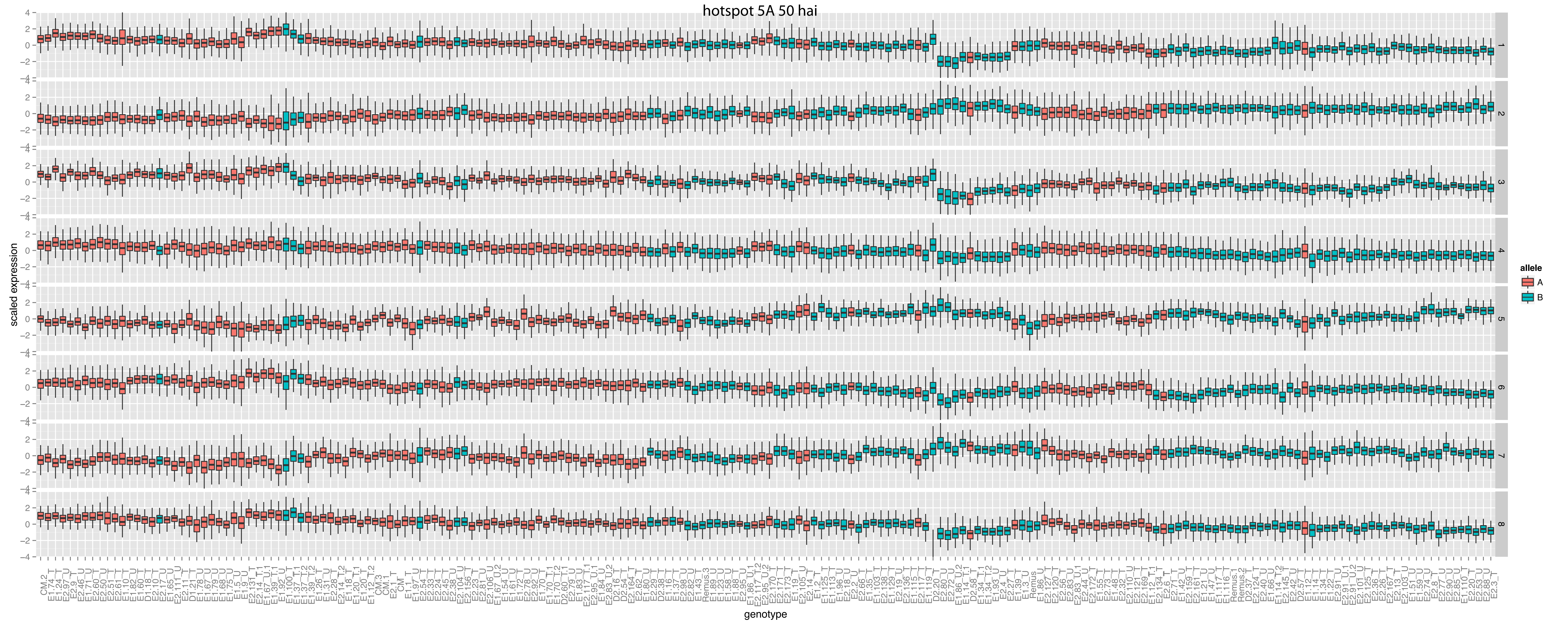
hotspot 5A 50 hai
scaled expression
genotype
allele
A
B
